# Advances in Immune Checkpoint Inhibitors for Cancer Treatment

**DOI:** 10.3390/cancers18111804

**Published:** 2026-06-01

**Authors:** Keqiang Chen, Feng Zhu, Xin Li, Teizo Yoshimura

**Affiliations:** Cancer Innovation Laboratory, Center for Cancer Research, National Cancer Institute at Frederick, Frederick, MD 21702, USA

**Keywords:** cancer immunotherapy, immune checkpoint proteins, immune checkpoint inhibitors, tumor microenvironment, emerging immune checkpoint proteins

## Abstract

The discovery of immune checkpoint proteins has marked a major breakthrough in cancer immunotherapy. During cancer development and progression, tumor cells exploit multiple immune checkpoint pathways to evade immune surveillance. Immune checkpoint inhibitors (ICIs) have been developed to restore antitumor immunity by reactivating immune cells that are functionally suppressed within the tumor microenvironment. This review provides a comprehensive overview of the regulation and therapeutic targeting of immune checkpoint proteins in cancer, covering both classical checkpoints, including PD-1, PD-L1, and CTLA-4, and emerging targets such as LAG-3, TIM-3, TIGIT, BTLA, SIRP-α, CD200, ILT4, and CD24. In addition, we summarize current clinical advances and the therapeutic potential of approved and investigational ICIs, examine the mechanisms underlying ICI resistance, and discuss future strategies to overcome resistance, highlighting the mechanisms of action and their implications for future cancer treatment.

## Simple Summary

Tumor cells evade attack by the immune system through immune checkpoint proteins, making these proteins important targets in cancer immunotherapy. By targeting them, immune checkpoint inhibitors (ICIs) help restore the body’s antitumor response by reactivating immune cells suppressed in the tumor microenvironment. This review summarizes well-known immune checkpoint proteins such as PD-1, PD-L1, and CTLA-4, as well as emerging checkpoints like LAG-3, TIM-3, TIGIT, BTLA, SIRP-α, CD200, ILT4, and CD24, together with recent clinical advances. It also explains why some patients develop resistance to these treatments and explores future strategies to improve their effectiveness in cancer therapy.

## 1. Introduction

Cancer immunotherapy is a therapeutic strategy that enhances or reactivates the body’s immune system to fight cancer [1]. It improves the ability of immune cells to recognize and eliminate malignant cells. Major immunotherapeutic approaches include immune checkpoint inhibitors (ICIs) [2], chimeric antigen receptor (CAR) T-cell therapy [3], cancer vaccines [4], cytokines [5], antitumor antibodies [6], and immunomodulators [7,8,9].

Over the past several years, ICI therapy has become a central component of modern cancer treatment. ICIs are monoclonal antibodies (mAbs) that interfere with immune checkpoint molecules to enhance antitumor immune responses. These molecules can be classified into two broad groups: classical immune checkpoint proteins, such as programmed cell death protein 1 (PD-1), programmed death ligand 1 (PD-L1), programmed death ligand 2 (PD-L2), and cytotoxic T-lymphocyte-associated antigen 4 (CTLA-4); and emerging immune checkpoint proteins, such as lymphocyte activation gene 3 (LAG-3), T-cell immunoglobulin and mucin domain 3 (TIM-3), T-cell immunoglobulin and ITIM domain (TIGIT), B and T lymphocyte attenuator (BTLA), signal regulatory protein alpha (SIRPα), CD200, immunoglobulin-like transcript 4 (ILT4), and CD24. Nevertheless, challenges remain, as primary nonresponse and acquired resistance to ICI therapy limit its efficacy. In this review, we summarize recent advances in ICIs, discuss their clinical applications, examine the mechanisms underlying ICI resistance, and highlight future strategies to overcome resistance in cancer treatment.

### Classical Immune Checkpoint Proteins

Classical immune checkpoint proteins, primarily CTLA-4 and PD-1/PD-L1, were the first to be identified and remain the most extensively studied. The inhibitory signaling pathways regulated by these proteins play essential roles in maintaining immune homeostasis and self-tolerance. However, tumor cells often hijack these pathways to suppress antitumor responses. The development of classical ICIs made it possible to block this tumor-mediated immunosuppression, thereby restoring T-cell function and enabling the elimination of cancer cells [10,11].

## 2. PD-1

### 2.1. Expression of PD-1 and Its Ligands

PD-1 was first identified in 1992 [12] and is primarily expressed on T cells. It is also detected on other immune cell types, including B cells, natural killer (NK) cells, and subsets of myeloid cells [12]. Notably, PD-1 expression has also been reported in various tumor cells, including melanoma, hepatocellular carcinoma (HCC), and non-small cell lung cancer (NSCLC) cells [13].

The ligands of PD-1 are PD-L1 [14] and PD-L2 [15], which were identified in 1999 and 2001, respectively. PD-L1 is expressed in inflamed tissues and in malignant cells in various tumor types, including lung cancer [16], bladder cancer [17], melanoma [18], breast cancer [19] and HCC [20]. Higher PD-L1 expression has been associated with more aggressive disease and poorer survival [21].

PD-L2 was first identified on macrophages and dendritic cells (DCs) [22] and is expressed in several human cancers, often overlapping with PD-L1 expression, including head and neck squamous cell carcinoma (HNSCC), lung squamous cell carcinoma (LUSC), renal cell carcinoma (RCC), pancreatic ductal adenocarcinoma (PDAC), and cervical cancer [11,12,13,14,15]. Similar to PD-L1, high PD-L2 expression has been correlated with poor clinical outcomes in several types of tumors [23,24,25].

### 2.2. PD-1-PD-L1/PD-L2 Signaling in Tumor Immune Evasion

The interaction between PD-1 on activated T cells and PD-L1 on tumor cells recruits phosphatases to the intracellular tail of PD-1, thereby suppressing T-cell activation. This signaling cascade induces T-cell exhaustion, reduces cytokine production, and inhibits T-cell proliferation, enabling tumor cells to evade immune surveillance [26,27].

Similarly, the interaction between PD-L2 on tumor cells and PD-1 on activated T cells suppresses T-cell proliferation, diminishes cytokine secretion, and promotes T cell exhaustion, thereby facilitating tumor evasion. Accordingly, PD-L2 may play an important role in cancers characterized by elevated PD-L2 levels [28].

PD-L1 and PD-L2 both suppress T-cell activation through the PD-1 pathway, but they differ in their expression pattern. PD-L1 is widely expressed by tumor cells in many malignancies, whereas increased PD-L2 expression has been observed in a more limited range of cancers, including HNSCC, esophageal adenocarcinoma, gastric cancer, prostate cancer, bladder cancer, salivary gland cancer (SGC), and colorectal cancer (CRC) [22,25,29].

Both ligands can also be expressed on immune cells; however, PD-L2 expression appears to be more closely associated with tumor-associated macrophages (TAMs). Some studies suggest that in CRC, PD-L2 may help maintain immune suppression when PD-L1 activity is reduced [30,31]. In addition, PD-L2 binds PD-1 with up to sixfold higher affinity than PD-L1 does [22].

### 2.3. ICIs Targeting PD-1 and PD-L1

Anti-PD-1/PD-L1 ICIs block PD-1 signaling, restore T-cell activity, and enhance antitumor immunity. As a result, they have become a major advance in cancer therapy [27]. Several FDA-approved anti-PD-1 antibodies are currently used for cancer treatment, including nivolumab (Opdivo), cemiplimab, and pembrolizumab (Keytruda). Pembrolizumab is approved for advanced melanoma and NSCLC [32], whereas nivolumab is used for HCC [33], stage III NSCLC [34], and metastatic CRC [35]. In addition, cemiplimab is approved for the treatment of metastatic cutaneous squamous cell carcinoma (CSCC) [36].

The FDA has also approved several anti-PD-L1 antibodies, including avelumab (Bavencio), durvalumab (Imfinzi), and atezolizumab (Tecentriq, MPDL3280A).

Atezolizumab, a humanized IgG1 mAb containing an engineered Fc fragment, enhances T-cell-mediated killing of cancer cells by blocking PD-L1 expressed on tumor cells [37]. It has been used in several malignancies, including metastatic bladder cancer [37], metastatic CRC [38], breast cancer [39,40,41], NSCLC [42,43,44], HCC [45], and prostate cancer [46]. Avelumab is a human IgG1 mAb that has been used to treat several cancers, including refractory metastatic urothelial carcinoma [47,48,49], gastric cancer [50], ovarian cancer [51], and metastatic CRC [52]. Durvalumab, another human IgG1 mAb, has been tested in urothelial carcinoma [53], NSCLC [54,55], small cell lung cancer [56,57,58,59], and biliary tract cancer [60]. Table 1 summarizes the clinical applications of ICIs targeting the PD-1 pathway.

### 2.4. ICIs Targeting PD-L2

Currently, ICIs targeting PD-L2 remain in the early stages of clinical development and are not yet available for routine clinical use. Only a few experimental PD-L2-targeting antibodies have been investigated in cancer models, including the murine mAb clone MEB123.3G2.038 (3G2). This antibody was generated using human PD-L2-Fc (amino acids 20–219) and human PD-L2-His (amino acids 1–219) fusion proteins as immunogens and was originally developed for PD-L2 immunohistochemistry (IHC) [86]. It shows high specificity for PD-L2 and is useful for detecting PD-L2 expression in tumor tissues. Although both PD-L1 and PD-L2 bind to PD-1, PD-L2 can also interact with repulsive guidance molecule b (RGMb). Blockade of the PD-L2-RGMb pathway has been shown to enhance antitumor responses in several mouse tumor models [87], suggesting a potentially effective immunotherapeutic strategy for patients who are unresponsive to anti-PD-1 immunotherapy.

Overall, these findings suggest that the PD-1/PD-L1/PD-L2 axis plays an important role in tumor immune evasion and disease progression. Although therapies directly targeting PD-L2 remain in early development, ICIs targeting this pathway have demonstrated significant clinical benefit in a variety of solid and hematological malignancies, highlighting their promise in cancer treatment.

## 3. CTLA-4

### 3.1. Expression of CTLA-4 and Its Ligands

CTLA-4 (CD152) was first identified by cDNA cloning in 1987 [88]. In humans, the *CTLA-4* gene is located on chromosome 2, whereas in mice it is found on chromosome 1. It encodes a 25 kDa cell-surface protein that is primarily expressed by T cells, especially activated CD4^+^ and CD8^+^ T cells, as well as regulatory T (Treg) cells [89]. Recent studies have reported CTLA-4 expression in TAMs, where it has been linked to M2 polarization and the development of an immunosuppressive tumor microenvironment [90]. CTLA-4 expression has also been detected in tumor cells from multiple cancer types, including melanoma, lung, ovarian, and breast cancer, CRC, sarcomas, neuroblastoma, and certain leukemia and lymphomas [91,92].

The ligands for CTLA-4, CD80 (B7-1) and CD86 (B7-2) are mainly expressed on antigen-presenting cells (APCs), such as activated DCs, monocytes, macrophages, and B cells [89,93,94]. Expression of CD80 and CD86 has also been found in several malignancies, including NSCLC, glioma, gastric carcinoma, pancreatic carcinoma and hematologic malignancies [95,96,97,98,99].

### 3.2. CTLA-4-CD80/CD86 Signaling in Tumorigenesis

CTLA-4 is a key immune checkpoint that binds CD80/CD86 and suppresses T-cell activation, thereby serving as a brake to maintain immune homeostasis [100]. CD28, a homolog of CTLA-4, also binds CD80/CD86, but in contrast delivers a costimulatory signal that cooperates with T-cell receptor (TCR) signaling to promote T-cell activation. Unlike CD28-mediated signaling, engagement of CTLA-4 by CD80/CD86 suppresses T-cell proliferation and activation [101,102]. In the TME, CD80/CD86 expressed on tumor cells can bind to CTLA-4 on T cells, thereby inhibiting T-cell activation, promoting T-cell anergy or apoptosis, and facilitating immune evasion rather than stimulating T cells through CD28 [103].

Within the TME, CTLA-4 is also expressed on tumor-infiltrating lymphocytes, Tregs, and tumor cells, while CD80 and CD86 are upregulated on APCs. CTLA-4 expressed on tumor cells can interact with CD80 on APCs, competing with T cells for ligand binding, thereby inhibiting T-cell activation, enhancing immune suppression, and reducing antitumor immunity [104,105].

### 3.3. ICIs Targeting CTLA-4

By targeting CTLA-4, anti-CTLA-4 antibodies disrupt its negative regulation on T-cell activation and thereby enhance immune responses against cancer cells [106]. Ipilimumab (YERVOY) is a human monoclonal IgG1 antibody that blocks CTLA-4, enabling T cells to more effectively attack cancer cells [107]. Ipilimumab has been used in the treatment of melanoma [107,108,109], NSCLC [110,111,112], renal cell carcinoma [113,114,115], HCC [116,117], CRC [118,119,120] and other types of cancers. Tremelimumab (IMJUDO) is another human CTLA-4-blocking monoclonal IgG2 antibody [121]. Tremelimumab has been approved to treat HCC [121,122,123] and NSCLC [124].

As discussed above, CTLA-4 is an inhibitory immune checkpoint that suppresses T-cell activation and contributes to tumor immune evasion. Therapeutic blockade of CTLA-4 with antibodies such as ipilimumab and tremelimumab can restore antitumor immunity and has demonstrated clinical benefit for multiple cancers.

#### Emerging ICIs

Emerging ICIs target novel immune checkpoint pathways, including LAG-3, TIM-3, TIGIT, BTLA, SIRPα, CD200, ILT4, and CD24 [125,126,127,128,129,130,131,132]. Beyond the well-characterized PD-1/PD-L1 and CTLA-4 axes, these pathways are expanding therapeutic options for patients with cancer. These emerging ICIs are at different clinical development stages (https://clinicaltrials.gov/): some, such as those targeting BTLA, SIRP-a, CD200, CD24, and ILT4, are in phase I/II trials, whereas others, including TIGIT, TIM-3, and LAG-3, have advanced to phase III trials. A phase III trial of LAG-3-targeted therapy showed positive results, leading to FDA approval of its combination with the PD-1 inhibitor nivolumab for patients with metastatic or unresectable melanoma [125,133,134,135]. However, phase III trials of TIGIT- and TIM-3-targeted therapies in lung cancer have failed [136,137], highlighting the need for further studies to better define and enhance their clinical efficacy. Accordingly, ICIs targeting these molecules are discussed below.

## 4. LAG-3

### 4.1. Expression of LAG-3 and Its Ligands

LAG-3 was first identified in 1990 and later recognized as an immune checkpoint receptor [138]. In humans, the *LAG-3* gene is located on chromosome 12 (12p13.32), whereas in mice it is located on chromosome 6. LAG-3 encodes a 70 kDa type I transmembrane protein that is primarily expressed on tumor-infiltrating lymphocytes (TILs), including CD8^+^ T cells, CD4^+^ T cells, and Tregs, but is generally absent from naïve T cells [139,140]. LAG-3 is also expressed by macrophages in the TME, particularly CD163^+^ M2-polarized TAMs [141].

The canonical ligand of LAG-3 is major histocompatibility complex class II (MHC-II). In addition, LAG-3 has been reported to interact with fibrinogen-like protein 1 (FGL1), galectin-3 (Gal-3), liver sinusoidal endothelial cell lectin (LSECtin), and α-synuclein [142]. MHC-II is aberrantly expressed in various solid and hematological malignancies, including melanoma, breast cancer, CRC, ovarian cancer, NSCLC, and prostate cancer [143]. FGL1 is highly expressed in several cancer types, including liver, lung, prostate, breast, colon, and kidney cancers [144]. LSECtin is expressed in several malignancies, including melanoma, breast cancer, and gastrointestinal cancer [145]. In addition, α-synuclein expression has been linked to several malignancies, including melanoma, neuroblastoma, medulloblastoma, glioblastoma, and ovarian cancer [146].

### 4.2. Interaction Between LAG-3 and MHC II in Tumorigenesis

It has been shown that binding of LAG-3 to MHC II negatively regulates T-cell activity, weakens antitumor immunity, and promotes tumor immune tolerance and [147,148,149]. Engagement of LAG-3 on T cells binds to MHC II on APCs and, in some cancers, on tumor cells, can drive T-cell exhaustion and contribute to immune evasion. This interaction interferes with TCR signaling, limits T-cell proliferation and cytokine production, and can enhance the suppressive activity of Tregs [149]. In the TME, high LAG-3 expression on TILs, particularly exhausted CD4^+^ and CD8^+^ T cells, dampens T cell-mediated antitumor immune responses and enables cancer cells to evade immune surveillance [150]. Accordingly, elevated LAG-3 expression on tumor-infiltrating T cells is frequently associated with tumor progression and poor prognosis, making it both a marker of advanced disease and a promising target for immunotherapy [151].

### 4.3. ICIs Targeting LAG-3

To date, several therapeutic agents targeting LAG-3 have been developed to enhance T cell-mediated antitumor immunity. These include anti-LAG-3 mAbs, such as relatlimab and favezelimab, as well as eftilagimod alpha (efti), a soluble LAG-3 fusion protein that modulates antigen-presenting cell function [133,134,135,152,153,154,155].

Relatlimab in combination with nivolumab has been approved for the treatment of unresectable or metastatic melanoma [133,134,135]. It has also been investigated in other solid tumors, including CRC. CRC is the third most commonly diagnosed cancer globally and the second leading cause of cancer-related death [156]. Fewer than 15% of nonmetastatic colon cancers exhibit mismatch repair deficiency (dMMR), which is associated with a hypermutated and highly immunogenic phenotype [157]. Recent studies have shown that dMMR tumors respond particularly well to ICI therapy. In a clinical study, nivolumab in combination with relatlimab was associated with high pathologic response rates among patients with locally advanced dMMR colon cancer [157].

Favezelimab (MK-4280) is a humanized mAb that blocks the interaction between LAG-3 and MHC-II and has been evaluated in clinical trials for CRC [155]. In addition, GSK2831781 selectively depletes activated LAG-3-expressing T cells and has been investigated as a potential treatment for ulcerative colitis, a disorder associated with an increased risk of CRC [158].

Efti promotes activation of APCs and CD4^+^ and CD8^+^ T cells and may help overcome resistance to PD-1/PD-L1 inhibitors. Clinical trials in several cancer types, including melanoma, lung cancer, and CRC, have shown promising results. In a clinical study of melanoma, pembrolizumab in combination with subcutaneous administration of efti has been shown to enhance CD8^+^ and CD4^+^ T-cell activation and increase levels of soluble biomarkers, particularly interferon-γ (IFN-γ), a Th1-associated cytokine. These findings suggest that efti is well tolerated in combination with pembrolizumab and may enhance antitumor activity [150].

**Figure 1:**
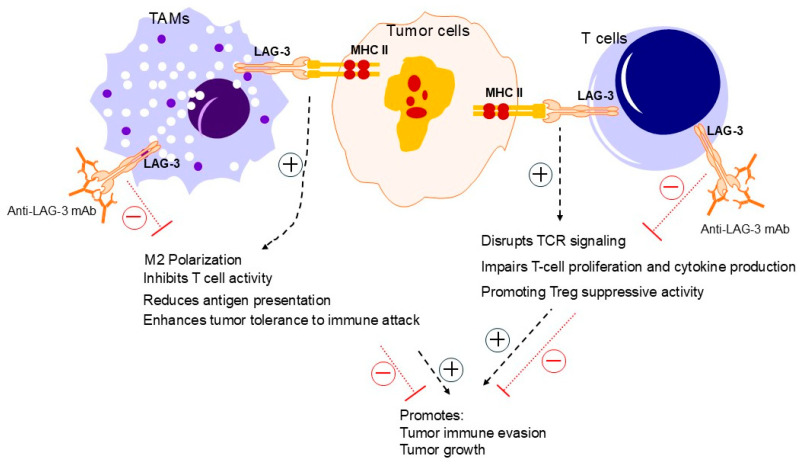
LAG-3 on TAMs or T cells interacts with MHC II on tumor cells, allowing cancer cells to evade immune surveillance. Anti-LAG-3 mAbs restore the immune system’s ability to fight cancer. The black dashed line indicates tumor-cell-induced immune checkpoint pathways, whereas the red dashed line indicates blockade of the immune checkpoint by anti-LAG-3 mAbs. Abbreviations: TAMs, tumor-associated macrophages; MHC II, major histocompatibility complex class II; mAb, monoclonal antibody; TCR, T-cell receptor; Treg, regulatory T cells.

NSCLC is the predominant type of lung cancer, accounting for about 85% of all cases worldwide, and is a major cause of cancer-related death [159]. Although ICIs targeting PD-1/PD-L1 are now routinely used as the first-line therapy, a subset of patients with NSCLC develops resistance. In patients with NSCLC resistant to PD-1/PD-L1 inhibitor, treatment with efti plus pembrolizumab showed preliminary evidence of antitumor activity in a clinical study [159]. Figure 1 summarizes the mechanisms by which LAG-3/MHC II interaction enables cancer cells to evade the immune system, and how blocking with anti-LAG-3 mAb restores the immune system.

Overall, LAG-3 is an inhibitory immune checkpoint receptor expressed on TILs, including T cells and Tregs, as well as macrophages/TAMs. By binding to ligands such as MHC II, it suppresses T-cell function, promotes tumor immune evasion, and is associated with poor prognosis. Combined LAG-3 and PD-1 blockade has shown clinical benefit, leading to FDA approval in advanced melanoma and supporting its potential in other cancers.

## 5. TIM-3

### 5.1. Expression of TIM-3 and Its Ligands

TIM-3, also known as CD366, was first identified in 2002 [160,161] and functions as an immune checkpoint receptor [125,162]. TIM-3 is primarily expressed on T cells, NK cells, DCs and macrophages [163,164,165]. M2-polarized TAMs, which are major contributors to the immunosuppressive TME, express high levels of TIM-3 [166]. In addition, TIM-3 is highly expressed on malignant cells in several cancers, including leukemia, glioblastoma, breast cancer, and CRC [162,167,168,169,170,171,172,173,174].

Known ligands of TIM-3 include Galectin-9 (Gal-9), phosphatidylserine (Ptdser), high-mobility group box-1 (HMGB-1), and carcinoembryonic antigen-related cell adhesion molecule-1 (CEACAM-1). Gal-9 and HMGB-1 are soluble, whereas PtdSer and CEACAM-1 are expressed on the cell surface of certain immune cells, such as T cells, NK cells, DCs, and macrophages, as well as on some cancer cells [162,175,176,177]. Among these ligands, Gal-9 has been reported to be prominently expressed by TAMs [178].

### 5.2. TIM-3 and Its Ligands in Tumorigenesis

The interaction between TIM-3 on TAMs and Gal-9 on tumor cells further increases TIM-3 expression and promotes macrophage polarization towards an immunosuppressive M2 phenotype. These cells express CD163, CD206, Arg-1, and IL-10 and often display high levels of TIM-3 [166,179]. TIM-3^+^ TAMs secrete IL-10 and TGF-β, thereby suppressing antitumor immune responses by inducing T-cell apoptosis and exhaustion, reducing the production of pro-inflammatory cytokines such as IFN-γ, and promoting an immunosuppressive TME [180]. These effects ultimately enhance angiogenesis and tumor growth [181,182].

Gal-9 on tumor cells also interacts with TIM-3 on T-cells, inducing TIM-3 phosphorylation at tyrosine residue Y265. This Gal-9/TIM-3 interaction initiates an inhibitory signaling pathway that promotes Th1 cell apoptosis, increases CD8^+^ T-cell exhaustion, and enhances the suppressive activity of Treg cells [183].

In a variety of tumors, TIM-3 expression is increased on TILs [184]. Compared with TIM-3^−^CD8^+^ TILs, TIM-3^+^CD8^+^ TILs exhibit significantly reduced cytotoxicity, with markedly decreased secretion of IL-2, TNF-α, IFN-γ, perforin, and granzyme B (GzmB), following engagement of TIM-3 by its ligands and activation of inhibitory signaling pathways [185]. TIM-3^+^CD4^+^ TILs also undergo increased cell death upon ligand binding [186]. In TIM-3^+^ FOXP3^+^ Treg cells, IL-10 production and sensitivity to IL-10 are increased, thereby promoting effector T-cell exhaustion and tumor progression [187].

### 5.3. Clinical Trials of ICIs Targeting TIM-3

Several anti-TIM-3 antibodies have entered clinical development [188]. Sabatolimab (MBG453) is a novel immunotherapeutic antibody targeting TIM-3 [189] and is being evaluated in clinical trials for acute myeloid leukemia (AML) and solid tumors [190,191]. TSR-022 (cobolimab) is a mAb that binds to TIM-3, thereby blocking its inhibitory function and promoting T-cell activation and antitumor immune responses [188]. M6903 is another fully humanized anti-TIM-3 antibody that blocks the interaction of TIM-3 with three of its ligands: PtdSer, CEACAM1, and Gal-9 [192].

Because TIM-3 is frequently co-expressed with PD-1 on TILs, anti-TIM-3 antibodies are often evaluated in combination with PD-1 or PD-L1 inhibitors in clinical trials. In one study, patients with NSCLC (*n* = 17) or advanced/metastatic melanoma (*n* = 16) received sabatolimab in combination with spartalizumab, an anti-PD-1 antibody. The combination was well tolerated, supporting further investigation of sabatolimab-based combination regimens across multiple tumor types [193]. In another study involving 219 patients, including those with ovarian cancer (17%) and CRC (7%), treatment with either sabatolimab (*n* = 133) or sabatolimab plus spartalizumab (*n* = 86) demonstrated preliminary evidence of antitumor activity [191].

**Figure 2:**
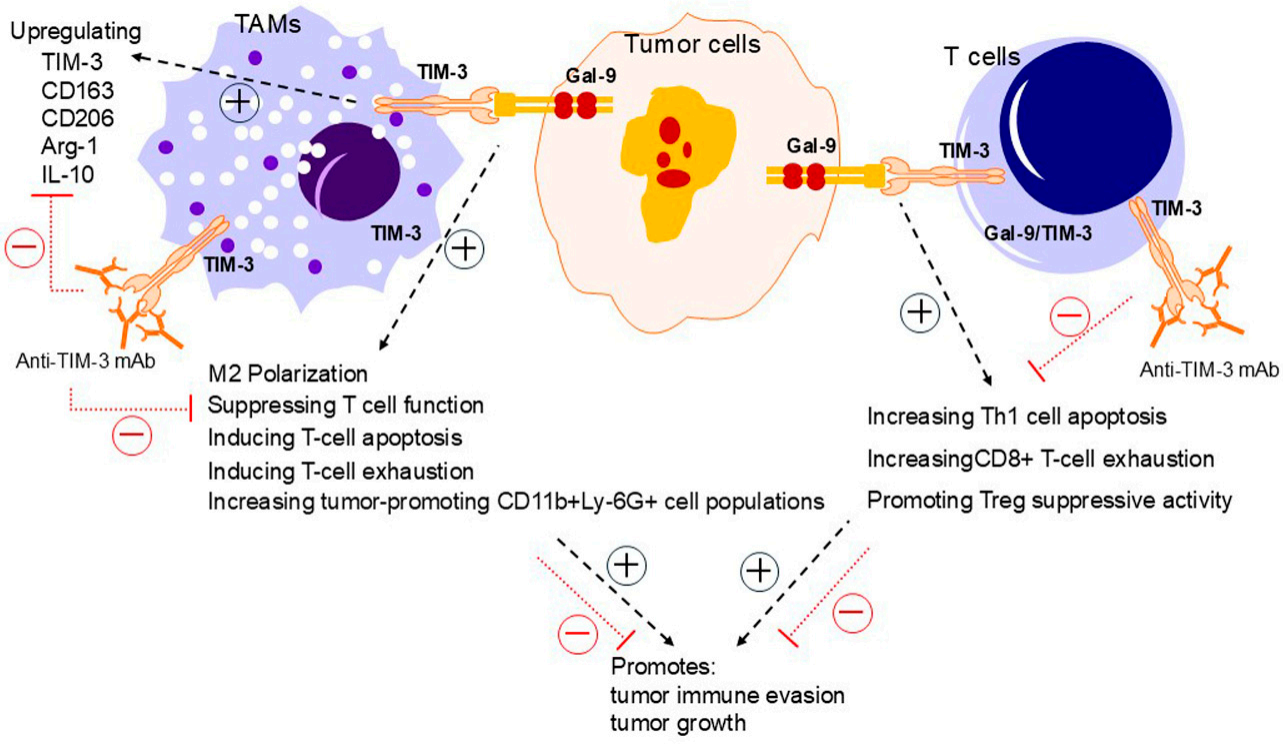
Gal-9 on tumor cells interacts with TIM3 on TAMs or T cells, promoting an immunosuppressive TME, enabling cancer cells to evade immune surveillance, and enhancing tumor progression. Anti-TIM-3 mAbs block Gal-9/TIM-3 signaling, reduce M2 macrophage infiltration and VEGF expression, and suppress tumor growth. The black dashed line indicates tumor-cell-induced immune checkpoint pathways, whereas the red dashed line indicates blockage of the immune checkpoint by anti-TIM-3 mAbs.

TIM-3 is considered an attractive therapeutic target in AML and myelodysplastic syndrome (MDS) because it is expressed in blasts and leukemic stem cells (LSCs), but not in normal hematopoietic stem cells. In clinical studies, adult patients with AML or MDS received sabatolimab in combination with hypomethylating agents (HMAs), a class of drugs that reverse DNA methylation and may reprogram malignant cells. The combination was well tolerated and showed antileukemic activity in treated patients [190]. Figure 2 illustrates the mechanisms by which Gal-9/TIM3 interactions enable cancer cells to evade immune surveillance and how anti-TIM-3 antibodies may block these effects.

Overall, TIM-3 is an inhibitory immune checkpoint receptor expressed on T cells, NK cells, DCs, macrophages/TAMs, and some tumor cells. Through ligands such as Gal-9, PtdSer, HMGB1, and CEACAM1, it promotes immunosuppression and tumor progression. Although TIM-3 blockade has failed in recent phase III lung cancer trials, further combination studies may improve its clinical efficacy.

## 6. TIGIT

### 6.1. Expression of TIGIT and Its Ligands

TIGIT, an immune checkpoint receptor also referred to as WUCAM, Vstm3, and VSIG9, was first identified in 2009 [194]. The *TIGIT* gene is located on chromosome 3q13.31 in humans [194]. TIGIT is primarily expressed on activated T cells, NK cells, Treg cells, and follicular helper T cells in humans [195]. In the TME, TIGIT is also expressed on M2-polarized TAMs, which are associated with higher tumor grades and poor prognosis [196]. M2 polarization of TAMs is accompanied by the expression of TIGIT and other co-regulatory receptors, including CD163, CD204, CD206, TIM-3, and LAG-3 [137]. Elevated TIGIT expression has also been reported in CRC tissues [197].

The ligands for TIGIT include CD155 (also known as PVR or Necl-5), CD112, and CD113 [195,198], which are expressed on tumor cells, DCs and macrophages [195,198,199]. TAMs also express CD155 [200].

### 6.2. TIGIT and Its Ligands in Tumorigenesis

Engagement of TIGIT by CD155 can induce APCs to increase IL-10 production and decrease IL-12 production, thereby suppressing T-cell and NK-cell activity [195].

CD155 expressed on tumor cells or TAMs interacts with TIGIT on immune cells, including NK cells and CD8^+^ T cells, leading to inhibition of the PI3K/AKT/mTOR pathway. This interaction directly suppresses NK-cell and CD8^+^ T-cell effector functions and enhances Treg activity, thereby allowing cancer cells to evade immune surveillance [195,201].

CD226 (DNAM-1) is a co-stimulatory receptor expressed on NK cells and activated CD4^+^ T cells, and its binding to CD155 promotes T-cell differentiation into Th1 and Th17 subsets. CD226 and TIGIT compete for binding to CD155 expressed on tumor cells. Therefore, increased TIGIT expression or decreased CD226 expression on immune cells constitutes a mechanism of cancer immune evasion [202,203,204].

### 6.3. Clinical Development of ICIs Targeting TIGIT

Currently, ICIs targeting TIGIT are still under clinical development. Several humanized anti-TIGIT antibodies have entered clinical trials, including tiragolumab and domvanalimab, which are being evaluated in lung cancer, as well as ociperlimab, which is being studied in combination with tislelizumab, an anti-PD-1 antibody, for the treatment of advanced solid tumors [205,206,207,208].

**Figure 3:**
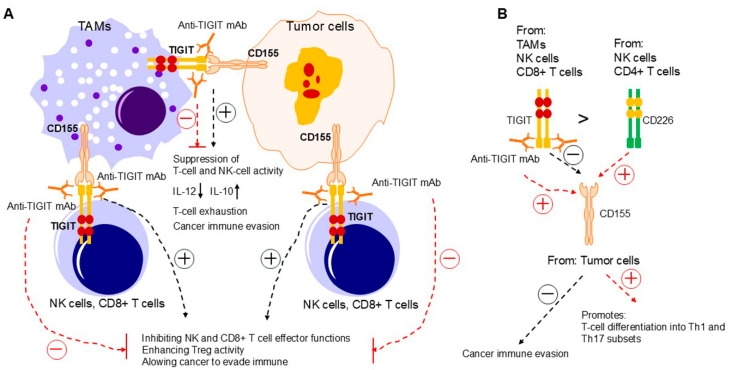
(**A**) CD155 on tumor cells interacts with TIGIT on TAMs or T-cells, and that TIGIT on T cells interacts with CD155 on TAMs or tumor cells. These interactions allow cancer cells to evade immune surveillance within the TME. The black dashed line indicates tumor-cell- induced immune checkpoint pathways, whereas the red dashed line indicates blockade of the immune checkpoint by anti-TIGIT mAbs. (**B**) CD226 is a stimulatory receptor that interacts with CD155, promoting T-cell differentiation into Th1 and Th17 subsets and maintaining the immune system’s ability to fight cancer. TIGIT competes with CD266 for binding to CD155, and elevated TIGIT expression represents a mechanism of cancer immune evasion. The red dashed line indicates the interaction between CD226 and CD155, whereas the black dashed line indicates competition by TIGIT for binding to CD155. Abbreviations: NK, natural killer; Th1, T helper 1; Th17, T helper 17.

TILs in both mice and humans have been shown to co-express TIGIT and PD-1 [209], as well as other inhibitory receptors such as TIM-3 and LAG-3 [195,210]. Blockade of either TIGIT or PD-1 alone does not significantly inhibit tumor growth in models such as murine colon cancer or human melanoma. However, combined blockade of TIGIT and PD-1 synergistically promotes complete tumor rejection and prolongs overall survival by enhancing the proliferation and effector function of antitumor CD8^+^ T cells, as well as the formation of protective memory T cells [208,211,212]. Figure 3A shows the mechanisms by which CD155/TIGIT interactions lead to cancer cell immune evasion. Figure 3B shows how TIGIT inhibitors promote the binding of CD155 to CD226.

Overall, TIGIT is an inhibitory immune checkpoint receptor expressed on activated T cells, NK cells, Tregs, and macrophages/TAMs. By binding CD155, CD112, and CD113, it promotes tumor immune evasion, partly through competition with CD226. Although TIGIT blockade has failed in recent phase III lung cancer trials, combination strategies, particularly with PD-1 blockade, may improve its clinical efficacy.

## 7. BTLA

### 7.1. Expression of BTLA and Its Ligands

BTLA, also known as CD272, is an important immune checkpoint molecule that plays a key role in the fine-tuning of immune responses, primarily by delivering inhibitory signals, although context-dependent costimulatory effects have also been reported [213]. BTLA is highly expressed on activated T cells and B cells, as well as macrophages and TAMs. Low levels of BTLA are detectable on immature DCs; however, its expression increases upon DC maturation [214]. BTLA has also been detected on various solid tumor cells, including NSCLC, melanoma, ovarian cancer, and gastric cancer [214].

BTLA ligands include herpes virus entry mediator (HVEM) and UL144 [214]. UL144 is a tumor necrosis factor receptor-like protein encoded by the UL144 gene of human cytomegalovirus (HCMV) and is an ortholog of HVEM [215]. HVEM is broadly expressed on immune cells, including T cells, B cells, NK cells, DCs, monocytes, TAMs and neutrophils [216], and serves as the primary ligand for the co-inhibitory receptor BTLA [217]. In addition, HVEM is expressed by multiple tumor types, including melanoma, lung cancer, breast cancer, esophageal cancer, and leukemia [218].

### 7.2. Function and the Role of BTLA and Its Ligands in Tumorigenesis

As an inhibitory immune checkpoint receptor, BTLA, expressed on tumor cells, binds to HVEM on TAMs and delivers inhibitory signals similar to those of the well-known PD-1/PD-L1 pathway, thereby promoting TAM polarization toward the M2 phenotype, which in turn suppresses T-cell responses and enhances tumor progression within the TME [219]. Tumor cells may also exploit the BTLA–HVEM axis to suppress innate immunity, thereby enabling immune evasion and promoting tumor angiogenesis, metastasis, and resistance to chemotherapy [213,214,220,221,222,223].

In addition, HVEM expressed on tumor cells interacts with BTLA on T cells, delivering inhibitory signals that inhibit T cell proliferation and cytokine production in the TME, thereby suppressing immune responses and promoting tumor progression [218].

### 7.3. Clinical Development of ICIs Targeting BTLA

**Figure 4:**
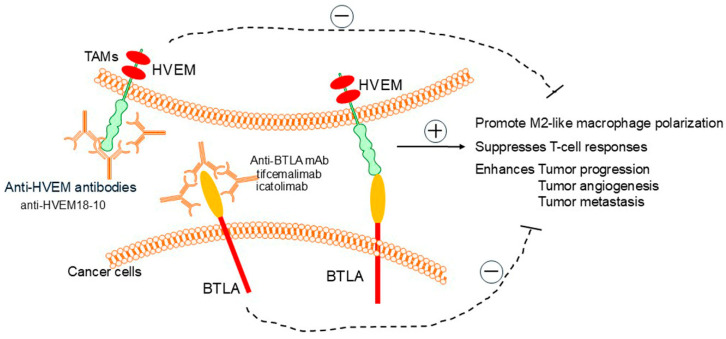
BTLA on tumor cells binds to HVEM on TAMs, triggering an inhibitory signal and promoting immunosuppression, and illustrates the clinical application of anti-BTLA or anti-HVEM mAbs. The black solid line indicates tumor-cell-induced immune checkpoint pathways, whereas the black dashed line indicates blockade of the immune checkpoint by anti-BTLA or anti-HVEM mAbs.

Anti-BTLA mAbs may exert immunomodulatory and antineoplastic effects by acting as ICIs, making BTLA a promising target for cancer immunotherapy [224,225,226,227]. To date, the anti-BTLA mAbs tifcemalimab and icatolimab have been evaluated in phase 1 studies in patients with relapsed or refractory lymphomas and advanced solid tumors, respectively [224]. In addition, anti-HVEM mAbs, such as anti-HVEM18-10, have been developed and evaluated in preclinical studies [228]. For example, treatment with anti-HVEM18-10 mAb enhanced T-cell responses against the lung cancer cell line NCIH2291 and increased the proliferation ratio and CD25 expression of CD4^+^ and CD8^+^ T cells, as well as production of TNF-α and IFN-γ [228]. Figure 4 illustrates the mechanisms by which the interaction between BTLA expressed on tumor cells and HVEM on TAMs induces immune suppression, as well as the clinical application of anti-BTLA or anti-HVEM mAbs.

Overall, BTLA is an inhibitory immune checkpoint receptor expressed on activated T cells, B cells, DCs, macrophages/TAMs, and some tumor cells. Through interaction with its ligand HVEM, it suppresses T-cell function and promotes TAM polarization, thereby contributing to tumor immune evasion. Although these therapies remain in early clinical development, BTLA-targeted therapies represent a promising strategy for cancer immunotherapy.

## 8. SIRPα

### 8.1. Expression of SIRPα and Its Ligand CD47

SIRPα, also known as CD172α, is an inhibitory immune checkpoint receptor primarily expressed on myeloid cells, including macrophages, monocytes, DCs, and neutrophils [128]. TAMs, particularly those with an immunosuppressive M2-like phenotype, express high levels of SIRPα within the TME, where high levels of SIRPα are expressed [229]. SIRPα is also expressed by specific subsets of CD8^+^ T cells [230].

CD47 is the primary ligand for SIRPα [231]. It is a ~50 kDa integral membrane protein with a heavily N-glycosylated N-terminal extracellular IgV-like domain, five transmembrane helices, and a short, variable C-terminal cytoplasmic tail [232]. CD47 is expressed on the surface of almost all healthy human cells [233] and is markedly overexpressed in nearly all types of human tumors, including breast cancer, ovarian cancer, colon cancer, bladder cancer, glioblastoma and leukemia [234].

### 8.2. SIRPα-CD47 Signaling in Tumorigenesis

The interaction between SIRPα on TAMs and CD47 on tumor cells induces phosphorylation of the immunoreceptor tyrosine-based inhibitory motifs (ITIMs) in the cytoplasmic domain of SIRPα. Phosphorylated SIRPα subsequently recruits the tyrosine phosphatases SHP-1 and SHP-2, thereby delivering a “do not eat me” signal that suppresses TAM-mediated phagocytosis of tumor cells [128,235].

### 8.3. Clinical Development of ICIs Targeting SIRPα-CD47

The SIRPα-CD47 axis has emerged as a promising therapeutic target in cancer immunotherapy. Several anti-SIRPα mAbs have been developed and evaluated in clinical trials. GS-0189 is a novel humanized anti-SIRPα mAb that has been investigated in combination with rituximab in patients with relapsed or refractory non-Hodgkin lymphoma [236]. DS-1103a, a newly developed anti-human SIRPα mAb, inhibits the SIRPα-CD47 interaction and enhances antibody-dependent cellular phagocytosis mediated by Dato-DXd and T-DXd against human cancer cells. It is currently being evaluated in clinical trials in patients with advanced solid tumors [237]. ADU-1805 is another humanized anti-SIRPα mAb that blocks the SIRPα–CD47 innate immune checkpoint pathway, thereby promoting antitumor immune responses [238]. In February 2023, the U.S. Food and Drug Administration (FDA) cleared ADU-1805 for safety and pharmacokinetics studies in adults with advanced solid tumors.

**Figure 5:**
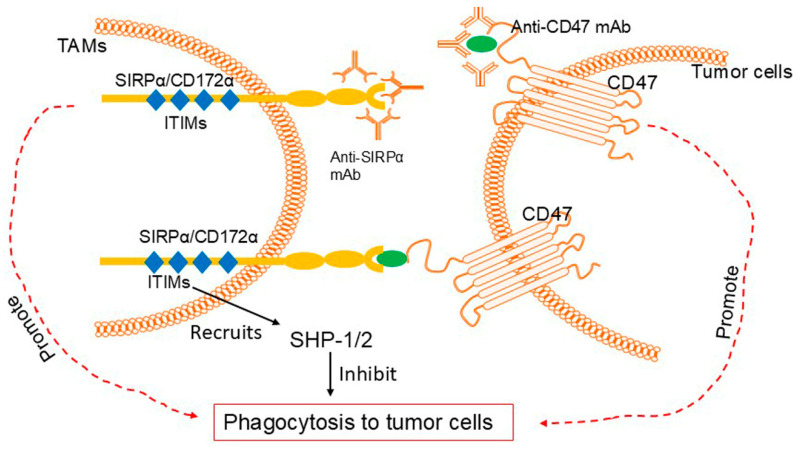
Interaction between SIRPα on TAMs and CD47 on tumor cells illustrates how anti-SIRPα or anti-CD47 mAbs block this interaction, thereby enhancing antibody-dependent cellular phagocytosis. The black solid line indicates tumor-cell-induced immune checkpoint pathways, whereas the red dashed line indicates blockade of the immune checkpoint by anti-CD47 or anti-SIRPα mAbs.

Anti-CD47 mAbs have also been developed to block the CD47-mediated “do not eat me” signal on tumor cells, thereby enabling macrophages to phagocytose and eliminate tumor cells. These antibodies include magrolimab (Hu5F9-G4), AO-176, and lemzoparlimab (AC001). In clinical studies, anti-CD47 antibodies have been shown to activate innate immunity and promote antitumor responses [239,240,241]. Figure 5 illustrates the mechanism by which SIRPα on TAMs interacts with CD47 on tumor cells and how anti-SIRPα or anti-CD47 mAbs block this interaction, thereby enhancing antibody-dependent cellular phagocytosis.

Overall, SIRPa is an inhibitory immune checkpoint receptor mainly expressed on myeloid cells, especially macrophages/TAMs. By binding to CD47, it delivers a “do not eat me” signal that inhibits phagocytosis and promotes tumor immune evasion.

## 9. CD200

### 9.1. Expression of CD200 and CD200R

CD200, also known as the OX-2 membrane glycoprotein, is an immune checkpoint molecule [130]. The gene encoding CD200 is located on chromosome 3 (3q12-13). Human and mouse CD200 share 77.6% homology at the protein level and 81.7% at the DNA level [242]. CD200 is expressed on various cell types, including B cells, DCs, activated T cells, endothelial cells and neuronal cells, as well as on the surface of many tumor cells in cancers such as breast cancer and melanoma [225,243].

CD200R, the receptor for CD200, is a type I transmembrane glycoprotein belonging to the immunoglobulin (Ig) superfamily. In humans, the CD200R gene spans 52 kb and consists of nine exons encoding a 348-amino-acid cell-surface protein [244,245]. CD200R is an inhibitory immune receptor that is highly expressed on myeloid cells, including macrophages and TAMs, myeloid-derived suppressor cells (MDSCs), DCs [246], neutrophils and microglia [247]. It is therefore considered an important immune checkpoint [248].

### 9.2. CD200-CD200R Signaling in Tumorigenesis

The interaction between CD200 on tumor cells and CD200R on TAMs suppresses M1-polarized antitumor activity and enhances M-CSF-mediated M2 macrophage polarization within the TME [249]. It also promotes the expansion of MDSCs and Tregs, which inhibit T cell activity [250], thereby enabling tumor cells to evade immune surveillance.

In AML, CD200 expressed on tumor cells binds to CD200R on NK cells, delivering inhibitory signals that suppress NK-cell cytotoxicity and cytotoxic T-cell functions [249]. This impairment of antitumor activity allows leukemic cells to evade immune surveillance, thereby promoting disease progression [249].

### 9.3. Clinical Development of ICIs Targeting CD200/CD200R

Because high CD200 expression on tumor cells can inhibit immune cell activity, blockade of the CD200-CD200R interaction with therapeutic antibodies targeting CD200 may enhance antitumor immune responses and represent a novel strategy for cancer treatment [251].

CD200 is expressed in human PDAC cell lines (BxPC3, MiaPaca2, and PANC-1), as well as in primary pancreatic epithelial cells and smooth muscle actin-positive stromal cells. CD200R expression is upregulated on CD11b^+^CD33^+^HLA-DR^lo/−^ MDSCs isolated from patients with PDAC. In vivo treatment with an anti-CD200 antibody limited tumor progression and significantly reduced intratumor MDSC populations [252].

The primary cause of relapses in AML is the persistence of LSCs [253]. CD200 is overexpressed in patients with AML, and the fully human monoclonal anti-CD200 antibody TTI-CD200 functions as an ICI and restores antileukemic immune responses in vitro and in vivo [253,254]. However, refractory pediatric acute lymphoblastic leukemia (ALL) remains a major clinical challenge. CD200^+^ ALL cells can be targeted by TTI-CD200 in vitro, and their numbers are reduced following TTI-CD200 therapy in vivo [254].

**Figure 6:**
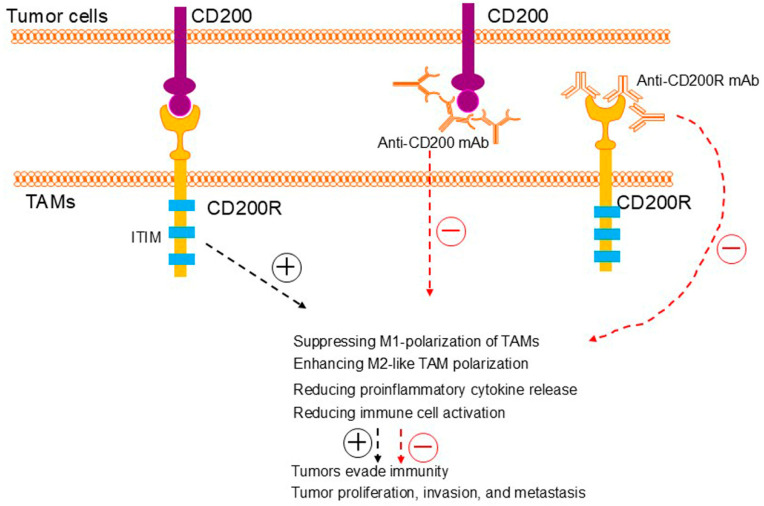
The interaction between CD200 on tumor cells and CD200R on TAMs promotes tumor immune evasion, and anti-CD200 mAbs limit tumor progression. The black dashed line indicates the tumor-cell-induced immune checkpoint pathways, whereas the red dashed line indicates blockade of the immune checkpoint by anti-CD200 or anti-CD200R mAbs.

Anti-CD200R mAbs, including anti-mouse CD200R (clone OX-110) and anti-human CD200R (clones OX-108 and EPR28088-189), are currently used only in laboratory research. Figure 6 illustrates how the interaction between CD200 on tumor cells and CD200R on TAMs promotes tumor immune escape and how anti-CD200 mAbs may limit tumor progression.

Overall, CD200 is an inhibitory immune checkpoint molecule expressed on various cell types, including some tumor cells, whereas its receptor, CD200R, is expressed on myeloid cells, including macrophages/TAMs, MDSCs, and DCs. The CD200-CD200R interaction promotes tumor immune evasion by enhancing M2 macrophage polarization and expanding immunosuppressive MDSCs and Tregs. Targeting this pathway may offer clinical benefit.

## 10. ILT4

### 10.1. Expression of ILT4 and Its Ligands

ILTs, also known as leukocyte immunoglobulin-like receptors (LILRs), CD85, or LILRBs, are encoded by a family of immunoreceptor genes located on human chromosome 19q13.4. This region, known as the leukocyte receptor complex, also contains genes encoding killer cell immunoglobulin-like receptors (KIRs), leukocyte-associated immunoglobulin-like receptors, NKp46, and the Fc alpha receptor (FcαR) [255].

ILT4, also known as LILRB2, leukocyte immunoglobulin-like receptor 2 (LIR2), or CD85d, was first identified in 1997 and is predominantly expressed on myeloid cells, including monocytes, macrophages, and DCs [256,257]. TAMs also express ILT4 [131]. In addition, ILT4 is highly expressed in several tumor types, including NSCLC, breast cancer, CRC and melanoma [258,259].

The primary ligands for ILT4 are MHC class I molecules, including HLA-A, HLA-B, and HLA-G [260]. Cells that express these ligands include tumor cells, trophoblasts, and certain immune cells, such as macrophages, TAMs, tolerogenic DCs, and MDSCs [261].

### 10.2. ILT4-HLA-G Signaling in Tumorigenesis

The interaction between ILT4 on tumor cells with HLA-G, which may be derived from TAMs within the TME, has been associated with activation of downstream signaling pathways, including the MAPK/ERK1/2 signaling axis. For example, increased ERK1/2 phosphorylation has been observed in NSCLC cells overexpressing ILT4, promoting cancer cell proliferation and motility [262].

ILT4 signaling can also activate the PI3K/AKT/mTOR pathway, thereby influencing tumor cell behavior and facilitating tumor progression. Activation of this pathway has been shown to increase expression of the coinhibitory molecule B7-H3 in NSCLC, contributing to tumor immune evasion and potentially promoting disease progression [263].

### 10.3. Clinical Development of ICIs Targeting ILT4

MK-4830 is a human mAb targeting ILT4 and is currently being evaluated in combination with anti-PD-1 antibodies in patients with advanced solid tumors [264]. NGM707 is a dual anti-ILT2/ILT4 humanized mAb being tested in clinical trials either as monotherapy or in combination with anti-PD-1 antibodies in patients with solid tumors. CHS-1000 and JTX-8064 are additional anti-ILT4 antibodies under development as candidates for cancer immunotherapy. Blocking ILT4 may help restore immune cell function and enhance the efficacy of other immunotherapies, such as anti-PD-1/PD-L1 antibodies [131,265].

**Figure 7:**
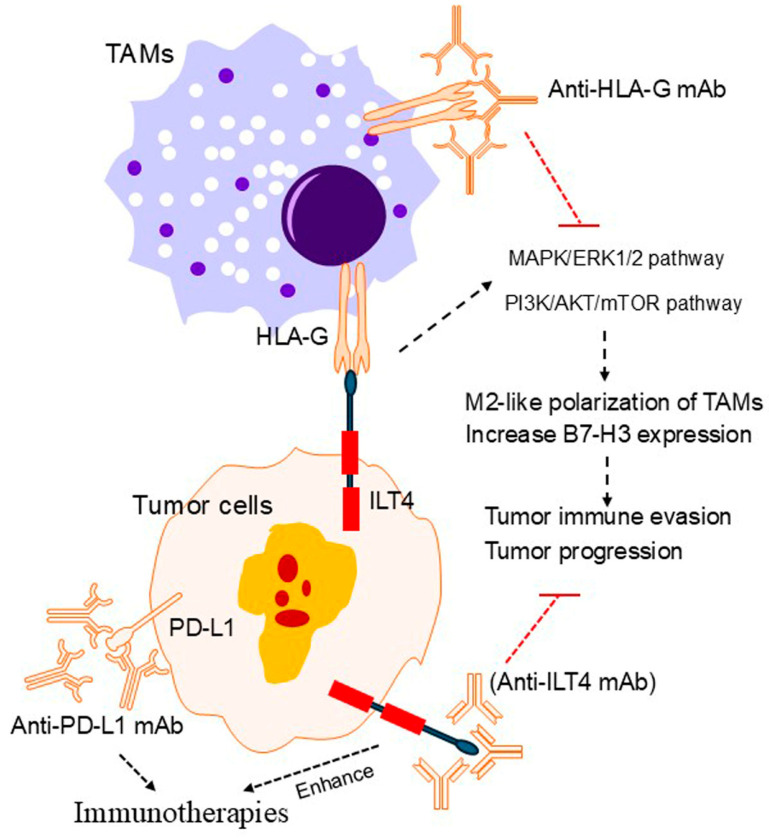
The interaction between ILT4 on tumor cells and HLA-G on TAMs promotes tumor immune evasion, and anti-ILT4 or anti-HLA-G mAbs inhibit tumor progression. The black dashed line indicates tumor-cell-induced immune checkpoint pathways, whereas the red dashed line indicates blockade of the immune checkpoint by anti-ILT4 or anti-HLA-G mAbs.

Anti-HLA-G mAbs, including 4H84, 87G, MEM-G/1, MEM-G/9, and 01G, have been developed to detect or inhibit human HLA-G; however, reports of their clinical application remain limited [266]. Figure 7 illustrates the mechanisms by which the interaction between ILT4 on tumor cells and HLA-G on TAMs promotes tumor immune escape, and anti-ILT4 or anti-HLA-G mAbs may limit tumor progression.

Overall, ILT4 is an inhibitory immune checkpoint receptor mainly expressed on myeloid cells, including macrophages/TAMs and DCs, as well as some tumor cells. By binding to MHC I molecules, it promotes tumor immune evasion and progression through activation of protumor signaling pathways. ILT4-targeted therapies, alone or in combination with PD-1 blockade, are under early clinical development.

## 11. CD24

### 11.1. Expression of CD24 and Its Receptor, Sialic Acid-Binding Immunoglobulin-like Lectin 10 (Siglec-10)

CD24 was first identified in 1978, and the mouse *Cd24* gene was cloned in 1990. Soon thereafter, the human CD24 gene was identified. Human CD24 is located on chromosome 6q21 and encodes a heavily glycosylated surface protein that is anchored to the plasma membrane via a glycosylphosphatidylinositol (GPI) linkage. CD24 is expressed on mature granulocytes, B cells, and tumor cells [129]. CD24 is also overexpressed in numerous solid tumors and hematological malignancies, including breast cancer, ovarian cancer, PDAC, lung cancer, gastrointestinal cancers, urogenital cancers, gliomas, B-cell lymphomas, and erythroleukemia [267,268]. CD24 is a functional ligand for Siglec-10 and is considered a novel innate immune checkpoint molecule [269].

Siglec-10 is primarily expressed on immune cells, including TAMs, B cells, and some T cells in the TME [269,270]. Siglec-10 is considered an innate immune inhibitory receptor [270,271].

### 11.2. CD24-Siglec-10 Signaling in Tumorigenesis

The interaction between overexpressed CD24 on tumor cells and Siglec-10 on TAMs triggers inhibitory signaling through the phosphatases SHP-1 and SHP-2. This interaction suppresses the phagocytosis of tumor cells by TAMs, promotes an M2-like immunosuppressive phenotype, and reduces inflammation as well as toll-like receptor (TLR)-mediated responses [272,273].

Overexpressed CD24 on tumor cells can also bind to Siglec-10 on T cells, thereby reducing T cell-mediated antitumor activity through inhibition of TCR signaling and phosphorylation of key kinases, such as Lck and ZAP-70 [274].

### 11.3. Clinical Development of ICIs Targeting CD24–Siglec-10 Interaction

IMM47, a humanized anti-CD24 mAb, has shown strong antitumor effects in human Siglec-10 transgenic mouse models, induces immune memory responses, and enhances the efficacy of anti-PD-1 antibodies, including tislelizumab, Opdivo, and Keytruda [272]. ONC-841 is another humanized mAb designed to block Siglec-10, thereby restoring the ability of immune cells to eliminate tumor cells.

In vitro studies using human breast cancer MCF-7 cells showed that macrophages preferentially phagocytose CD24^−/−^ MCF-7 cells compared with wild-type MCF-7 cells, and that anti-CD24 antibody treatment enhances macrophage-mediated phagocytosis of MCF-7 cells. In addition, Siglec-10^−/−^ macrophages exhibit increased phagocytosis of MCF-7 cells [272].

**Figure 8:**
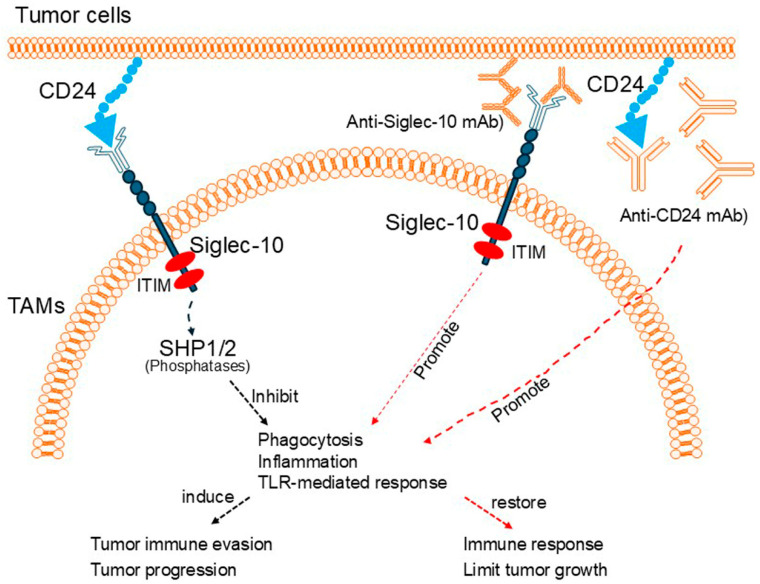
The interaction between CD24 on tumor cells and Siglec-10 on TAMs promotes tumor immune evasion, and anti-CD24 or anti-Siglec-10 mAbs inhibit tumor progression. The black dashed line indicates the tumor-cell-induced immune checkpoint pathways, whereas the red dashed line indicates blockade of the immune checkpoint by anti-CD24 or anti-Siglec-10 mAbs.

ONC-841 entered a phase 1 first-in-human clinical trial in 2024. Preclinical studies showed that ONC-841 promoted antitumor immunity in mouse models. Figure 8 illustrates the mechanisms by which the interaction between CD24 on tumor cells and Siglec-10 on TAMs drives tumor immune escape, and how anti-CD24 or anti-Siglec-10 mAbs may limit tumor progression.

Overall, CD24 is an inhibitory immune checkpoint molecule that is often overexpressed in cancer and binds Siglec-10 on immune cells to suppress antitumor immunity. Blockade of the CD24–Siglec-10 axis can restore immune attack on tumors, and early therapies such as IMM47 and ONC-841 have shown promising preclinical results, with ONC-841 entering phase 1 trials in 2024.

Clinical applications of emerging ICIs are summarized in Table 2.

#### Challenges and Prospects

A growing body of evidence has demonstrated that ICIs, particularly those targeting PD-1/PD-L1, CTLA-4, and LAG-3, have transformed the treatment landscape for several malignancies, whether used alone or in combination, by offering durable responses where traditional treatments often fail. However, the development and clinical application of ICIs still face several major challenges.

Studies have shown that patient responses to ICIs are highly variable, and it remains difficult to predict which patients will benefit from treatment. Although many patients benefit from anti-PD-1/PD-L1 or anti-CTLA-4 therapy, response rates vary dramatically depending on treatment modality (i.e., monotherapy versus combination therapy) and cancer types. For example, in one clinical study, patients with advanced non-colorectal cancer had a 34.3% response rate when treated with anti-PD-1 pembrolizumab [286], whereas in another study, patients with refractory triple-negative breast cancer achieved a 50% response rate following anti-PD-1 treatment [287].

Mechanistic studies have shown that multiple factors can contribute to primary nonresponse or acquired resistance to ICI therapy and may even accelerate tumor growth. Low tumor mutation burden (TMB) is one major cause of poor response, whereas patients with DNA mismatch repair deficiency, which often leads to microsatellite instability-high (MSI-H), or with defects in DNA repair genes involved in homologous recombination (HR) and nucleotide excision repair (NER), are more likely to benefit from ICIs [286]. On the other hand, certain mutations, such as those in p53, RAC1, and EGFR, can prevent T-cell infiltration and create an immunologically “cold” tumor microenvironment, thereby reducing the efficacy of ICI therapy [288,289,290]. T-cell exhaustion caused by chronic inflammation-mediated epigenetic alterations may represent another common mechanism of ICI resistance [291]. Similarly, defects in IFN-γ signaling and activation of oncogenic pathways, for example, the Wnt/β-catenin pathway, are associated with immune exclusion, thereby rendering tumor cells unresponsive to ICIs [292,293].

In addition, immune-related adverse events (irAEs) pose significant risks and require vigilant monitoring and management, as some can be severe or life-threatening. Caution is necessary when evaluating patients for IC therapy because irAEs may occur, including ICI-induced myocarditis and myositis, which can be life-threatening in some patients [294]. Patients with pre-existing autoimmune disease are also at increased risk of irAEs [295].

Lastly, the identification and validation of novel immune checkpoint targets require deeper insight into immune regulation, while high development costs, complex clinical trial designs, and the lack of reliable biomarkers hinder rapid progress.

Overall, integrated efforts across immunology, genomics, and clinical research will be instrumental in overcoming these challenges. Combination therapies and strategies to turn “cold” tumors “hot” are urgently needed to improve the success of ICI treatment [296]. As our understanding of immune regulation continues to deepen, the development of next-generation ICIs is expected to become more precise, safer, and more effective, thereby expanding therapeutic opportunities in cancer treatment.

## 12. Conclusions

Immune checkpoint proteins function as key mediators in tumor immune evasion, and the blockade of immune checkpoint pathways has reshaped the landscape of cancer treatment. While classical immune checkpoints targeting PD-1, PD-L1, and CTLA-4 have demonstrated significant clinical benefits, a growing number of emerging checkpoints, including LAG-3, TIM-3, TIGIT, BTLA, SIRP-α, CD200, ILT4, and CD24, open new avenues for enhancing antitumor immunity. Continued advances in understanding the mechanisms and crosstalk of these pathways will be invaluable for improving the efficacy of ICIs and overcoming resistance. Overall, ongoing advances in ICIs highlight their potential to enable more precise, effective and durable cancer immunotherapy strategies in the future.

## Figures and Tables

**Table 1:** Clinical applications of ICIs targeting PD-1/PD-L1.

Target	Name	Clinical Application
PD-1	Pembrolizumab (KEYTRUDA^®^) (IgG4, human)	Melanoma [32], Lung cancer [61,62], HNSCC [63], Prostate cancer [64], Esophageal cancer [65], Hodgkin lymphoma [66].
	Nivolumab (OPDIVO^®^) (IgG4, human)	Lung cancer [34], Hepatocellular carcinoma [33], Head and neck squamous cell carcinoma [67], Colorectal cancer [35], Melanoma [68], Renal cell carcinoma [69], Urothelial cancers [70].
	Cemiplimab (LIBTAYO^®^) (IgG4, human)	Cervical cancer [71], Lung cancer [72], Cutaneous squamous cell carcinoma [73,74].
PD-L1	Atezolizumab (TECENTRIQ^®^) (IgG1, human)	Hepatocellular carcinoma [75], Lung cancer [43,76], Breast cancer [39,40], Colorectal cancer [38].
	Avelumab (BAVENCIO^®^) (IgG1, human)	Urothelial carcinoma [48,77], Renal-cell carcinoma [78], Gastric cancer [50], Ovarian cancer [79], Merkel cell carcinoma [80].
	Duravulumab (IMFINZI^®^) (IgG1, human)	Lung cancer [54,55], Endometrial cancer [81], Hepatocellular carcinoma [82], Biliary tract cancer [83], Bladder cancer [84], Cervical cancer [85].

**Table 2:** Clinical applications of ICIs targeting Emerging Immune Checkpoints.

Target	Name	Clinical Trial
LAG-3	Eftilagimod alpha	Head and neck squamous cell carcinoma [149], Breast cancer [275,276], Melanoma [150].
	Relatlimab	Melanoma [133,277,278].
	Favezelimab	Colorectal cancer [155,279], Lung cancer [280].
TIM-3	Sabatolimab	Lung cancer [193], Leukemia [190,281,282].
	TSR-022	Leukemia [283].
TIGIT	Tiragolumab	Lung cancer [205,206], Hepatocellular carcinoma [284], Cervical cancer [285].
	Ociperlimab	Advanced solid tumors [207].
	Domvanalimab	Lung cancer [208]
BTLA	Tifcemalimab	Relapsed/refractory lymphomas [224]
	Icatolimab	Advanced solid tumors [224]
SIRPα	GS-0189	Non-Hodgkin lymphoma [236]
	DS-1103a	Advanced solid tumors [237]
CD200 ILT4	ADU-1805	Clinical trials for advanced solid tumors
	TTI-CD200	Preclinical studies for AML and ALL [227,228]
	MK-4830	Advanced solid tumors [236]
	NGM707	Solid tumors [237,238]
	CHS-1000	Solid tumors [249]
	JTX-8064	Solid tumors [238]

## Data Availability

No new data were generated in this study. All data discussed in this article are available found in the cited literature.
